# Factors associated with risk sexual behaviours of HIV/STDs infection among university students in Henan, China: a cross-sectional study

**DOI:** 10.1186/s12978-021-01219-3

**Published:** 2021-08-18

**Authors:** Xiaoan Du, Ling Zhang, Hong Luo, Wenlong Rong, Xianxin Meng, Hang Yu, Xiaodong Tan

**Affiliations:** 1 School of Health Sciences, Wuhan University, Wuhan, 430071 China; 2 North China University of Water Resources and Electric Power, Zhengzhou, 450000 China; 3 School of Health and Nursing, Wuchang University of Technology, Wuhan, 430223 China

**Keywords:** University students, Risk sexual behaviours, Condom use, Multiple sexual partners, Sexual debut

## Abstract

**Background:**

This study aimed to identify factors associated with risk sexual behaviours and target high-risk groups at risk of HIV/STDs infection among university students.

**Methods:**

The cross-sectional study was conducted from 1 November to 31 December 2020 in one university, located in Henan Province. A total of 1602 individuals who reported having ever had sex were analyzed as the subjects of this study. Descriptive analysis and multivariable logistic regression analysis were applied for this study to assess factors associated with risk sexual behaviours among university students.

**Results:**

University students who reported having ever had sex accounted for about 9%, with an average age of 19.37 ± 1.03. Of them, having multiple sexual partners and inconsistent condom use during the last 6 months were 37.3% and 35%, respectively. Over 50% of participants had their sexual debut before the age of 18. Bisexual students (AOR = 0.27; 95% CI 0.16, 0.44) and those who lived on over 3000 Yuan per month (AOR = 0.50; 95% CI 0.28, 0.91) were consistently less likely to engage in condom use during the last 6 months. University students who were from high-grade (AOR = 1.56; 95% CI 1.12, 2.18 for sophomore; AOR = 1.84; 95% CI 1.28, 2.65 for junior; AOR = 2.07; 95% CI 1.38, 3.11 for senior), who lived on over 3,000 Yuan per month (AOR = 4.19; 95% CI 2.17, 8.11) or who reported being homosexual (AOR = 3.92; 95% CI 2.17, 7.06) and bisexual (AOR = 33.22; 95% CI 13.11, 84.15) were more likely to have multiple sexual relationships. University students who had sexual debut before the age of 18 were more likely to engage in risk sexual behaviours.

**Conclusions:**

The prevalence of sexual activity among Chinese university students is generally low, but risk sexual behaviours are of considerable concern. University students with higher living expenses, who are not heterosexual and who are younger at first sexual intercourse tend to engage in risk sexual behaviours. The scale-up of intervention is the need to prevent the expansion of the HIV epidemic among young students.

## Plain Language Summary

The HIV epidemic among university students is considerable, with the tendency of the high-risk population towards the general population. University students at risk of HIV/STDs infection tend to engage in risk sexual behaviours and were targeted for assessing factors corelated with their risk sexual behaviours in this study. Participants who reported having had sexual relations were selected. Information on the practice of risk sexual behaviours including multiple sexual partners and inconsistent condom use were collected and analyzed using a multivariable logistic regression model.

Bisexual students were consistently less likely to use condoms within the last 6 months. University students from high-grade, who lived on over 3000 Yuan monthly, homosexual students and bisexual students were more likely to have multiple sexual relationships. University students who had sexual debut before the age of 18 were more likely to engage in risk sexual behaviours.

In conclusion, University students with high cost of living per month, non-heterosexual, who are younger at first sexual intercourse tend to engage in risk sexual behaviours. Intensified intervention is the need to prevent the expansion of the HIV epidemic amongst young students.

## Introduction

Acquired immune deficiency syndrome (AIDS) is a disease with highly mortality caused by infection with the human immunodeficiency virus (HIV). There are as yet no effective medicines to cure HIV infection worldwide. Instead, intervention for HIV infection is designed as the most effective means of preventing the spread of the HIV epidemic. Currently, the HIV epidemic has become a pervasive public health concern. Statistics from the Joint United Nations Program on HIV/AIDS (UNAIDS) show an estimated 38 (31.6–44.5) million people living with HIV (PLWH) and 1.7 million newly HIV infections in 2019 [1]; National Health Commission of the People’s Republic of China reported an estimated 0.96 million infections at the end of 2019 [2]. When compared to other countries, the overall HIV epidemic in China is generally at a low level, while the HIV epidemic in specific populations shows a higher level with the feature of sexual transmission as the main mode of transmission [3]. HIV can be widely transmitted through unprotected sex, which includes unprotected anal intercourse (UAI), unprotected vaginal intercourse (UVI) and multiple sexual partnerships [4]. The specific sexual practices of the gay community make them the most susceptible to HIV infection. National surveillance data showed that about 8 out of every 100 men who have sex with men (MSM) were at high risk of becoming infected with HIV [5]. From 2011 to 2015, the number of university students in China who contracted HIV jumped by 35% annually [6].

Although only 4% of male university students in China had sex with men [7, 8], data showed that the rate of HIV infection among male university students who had sex with other men raised from 58.5% in 2008 to 82% in 2014 [9]. In addition, the prevalence of other sexual transmitted diseases (STDs) among the population at high risk has become a general concern. Sentinel surveillance data from 2010 to 2015 in China indicated that rates of syphilis infection remained high among male attendees at the STD clinic and among MSM [10]. Syphilis was significantly related to HIV infection in most research on MSM [11, 12]. Nevertheless, the at-risk population, including MSM or men who had sex with women and men (MSWM), may contribute to the spread of HIV infection among the general population [13, 14].

Many factors are associated with the occurrence of risk sexual behaviours, which in turn contribute to the HIV/STDs epidemic among university students. With the mutual convergence of western liberalism and traditional Chinese culture, changes in sexual attitude [15], the lack of parental monitoring and reduced academic pressure [16], most Chinese university students who should be sexually active at early adolescence initiated their first sexual act during the college life [16, 17]. In 2017, however, a self-administered questionnaire survey from 15 provinces in China found that 24.2% of university students had engaged in sexual activity under the age of 18 [18]. When compared to document issued that the awareness rate of HIV/AIDS-related knowledge among young students will reach over 95% [19], the knowledge of HIV/sexually transmitted diseases (STDs) is limited or insufficient, especially at-risk groups vulnerable to HIV/STDs infection, engaging in high-risk sexual behaviours [8, 20, 21]. However, a prior study indicated that a high level of HIV/AIDS knowledge may access high-risk sexual intercourse [22], including inconsistent condom use, multiple sexual partners during the last 6 months and having sex with the same sex [23, 24]. Many studies indicated that university students who reported being homosexual or bisexual were associated with their high-risk sexual intercourse and related factors may be related to the access to social media such as apps searching for sexual partners [25–27].

The government, together with the Ministry of Education and other departments, has made efforts to control the development of the HIV epidemic in China [28]. But implementing HIV prevention services for university students has hardly any effect. For instance, only 7.3% of male participants had HIV testing, according to a study conducted in 2019 [22]. In 2018, a study of 222 universities across China showed that about 63% of university students were willing to take an HIV testing [29]. This may due to anonymous HIV testing services not available on a large scale in major universities in all regions of China, except for pilot service work conducted over 40 universities in 2018 [30].

In the age of the information explosion, university students sensitive to sex-related messages have witnessed a change in sexual attitudes that has led to a change in sexual behaviours. Henan Province, located in central China, has more than 50,000 PLWH diagnosed in 2018 [3]. The number of reported HIV cases among young students aged 15–24 years has risen from 65.8% in 2004 to 81.4% in 2019, with all routes of infection dominated by same-sex transmission [31]. Many articles studied on sexual knowledge, sexual attitude, sexual behaviours and the status of the HIV epidemic among young students in Henan have been published, but there are few articles conducted on factors associated with risk sexual behaviours. So, this study, based on data collected from one university in Henan, aims to: (a) describe demographic characteristics, awareness of HIV/AIDS-related knowledge, utilization of HIV prevention services among university students and; (b) identify associated factors among university students including condom use, the number of sexual partner and; (c) explore how to measure promising approaches to better preventing risk sexual behaviours among young students.

## Material and methods

### Participants and samples

The study is based on a cross-sectional survey conducted in November 2020. Approximately 17,678 male and female university students in one comprehensive university in Henan Province were purposefully sampled to ensure an adequate representation of students from freshmen to senior. With the permission and help of the Student Union of the departments, the study participants were recruited to complete an online questionnaire by scanning the WeChat (a Chinese cross-platform communication tool for sending voice, pictures, video and text over the mobile network) QR code. To study the factors associated with risk sexual behaviors among university students, the remaining 1602 samples who reported having ever had sexual intercourse met the study’s eligibility criteria. All the study participants were informed of the purpose of this study.

### Study variables and measurements

Data were collected through an online, anonymous, structured questionnaire, which is adapted from the “Chinese AIDS Sentinel Testing Implementation Plan”. The questionnaire consists of three indicators, involving demographic characteristics of the study participants, information on HIV/AIDS and practices of sexual behaviours. The participants were required to complete basic personal information regarding gender, grade, ethnicity, marital status, monthly income and sexual orientation. Information on HIV/AIDS included HIV/AIDS-related knowledge, HIV prevention services and self-perceived risk of HIV infection. HIV/AIDS-related knowledge was measured by 9 items with the answers “True, False, and Do not know”, as stratified by male and female students. For each item, the participants scored one point when they answered the item correctly or zero points if they answered the item incorrectly (including the “Do not know” answer). The total score of the HIV/AIDS-related knowledge was 9, with six and above indicating having sufficient HIV/AIDS knowledge; otherwise, score less than six meaning having insufficient HIV/AIDS-related knowledge. Practices of sexual behaviours referred to risk sexual behaviours, including consistent or inconsistent condom use, having single or multiple sexual partners. As the independent variables, demographic characteristics, level of HIV/AIDS-related knowledge, self-perceived risk of HIV infection and age of first sexual debut were included in the regression model.

### Data collection

Data were collected online via the Chinese professional survey website Wenjuanxing between 1 and 30 November 2020. All potential participants scanned the WeChat QR code to fill in the questionnaire and to successfully submit it when all the items were completed. Students were allowed to spare their leisure time completing the questionnaire anonymously, with a time limit of 30 min. Once the questionnaire had been submitted, the participants would be entered in a random draw as an reward.

### Statistical methods

Absolute number and percentage were descriptively applied for categorical variables, with mean and standard deviation (SD) for continuous variables. Comparisons of demographic variables, HIV/AIDS-related knowledge, utilization of HIV prevention services between the groups (male students vs female students) were performed using the Chi-square test. T-test and analysis of variance (ANOVA) were used to identify differences in HIV/AIDS-related knowledge scores between groups. Multivariable logistic regression analysis was performed to estimate factors significantly associated with youth sexual behaviours, including demographics, level of HIV/AIDS-related knowledge, perception of risk of HIV/STDs infection, utilization of HIV prevention programs. All statistical analyses were conducted using SPSS version 25.0. Statistical significance was accepted at the P values < 0.05 level.

## Results

### Demographic characteristics

The largest proportion of university students who ever had had sexual intercourse was sophomores at 33.4% (535/1602), while the lowest was at 17% (284/1602). The proportion of university students having sex progressively rose from freshmen to senior. Sexually active male and female students accounted for about 11% and 4% of the survey sample, respectively. The overwhelming majority of sexually active students were the Han (1449/1602), unmarried (1539/1602). Ethnic minorities were more sexually active than the Han. 42% of university students who lived on more than 3000 Yuan were more likely to engage in sexual intercourse. Around 60% (962/1602) of sexually active students lived with between 1000 Yuan and 2000 Yuan per month. Over 90% (1461/1602) of sexually active students were sufficiently knowledgeable about HIV/AIDS. University students who had HIV testing and received HIV prevention services were more likely to engage in sexual intercourse, compared to those who never had HIV testing and utilize HIV prevention services (Table 1).

**Table 1:** Comparison of characteristics of sexually active students among the sample of the respondents (N = 17,678)

Variables	Category	Ever had sexual intercourse		Frequency (%)	*P*
		Yes	No		
Grade	Freshmen	383	6009	6.0	< 0.001
	Sophomore	535	5205	9.3	
	Junior	400	3453	10.4	
	Senior	284	1409	16.8	
Gender	Male	1368	10,984	11.1	< 0.001
	Female	234	5092	4.4	
Ethnicity	Han	1449	15,185	8.7	< 0.001
	Minority	153	891	14.7	
Marriage	Unmarried	1539	15,970	8.8	< 0.001
	Married	63	106	37.3	
Monthly income	Under 1000	383	5774	6.2	< 0.001
	1000 ~ 2000	962	9534	9.2	
	2001 ~ 3000	155	627	19.8	
	Above 3000	102	141	42.0	
Awareness of HIV knowledge					
Insufficient		141	3259	4.1	< 0.001
Sufficient		1461	12,817	10.2	
HIV testing					
Yes		241	1502	13.8	< 0.001
No		1361	14,574	8.5	
Utilization of HIV prevention services					
Yes		900	8234	9.9	< 0.001
No		702	7842	8.2	

### HIV/AIDS-related knowledge

In total, nine items assessing the level of HIV/AIDS-related knowledge for participants are presented in Table 2. Only 64% of male participants were aware that HIV prevalence is increasing among adolescents, with sexual transmission among MSM being the primary mode of transmission. Over 90% of participants believed they should seek HIV testing and counselling after high-risk behaviours (needle sharing, unsafe sex, etc.). Moreover, there is a significant difference between male and female participants in the fact that consistent use of condoms may reduce HIV infection and sexual transmission.

**Table 2:** Awareness of HIV/AIDS-related knowledge among male and female students (n = 1602)

Statements	Total (%)	Male (%)	Female (%)	*χ*^2^	*P*
1. Do you think of HIV/AIDS as an incurable infection (yes)	1310 (81.8)	1138 (83.2)	172 (73.5)	12.569	< 0.001
2. Do you agree that the main mode of transmission is male homosexual sex, followed by heterosexual sex when the HIV epidemic is growing rapidly among young students in China (yes)	1023 (63.9)	873 (63.8)	150 (64.1)	0.007	0.933
3. Can you tell if someone is infected with HIV/AIDS by their appearance (no)	1390 (86.8)	1191 (87.1)	199 (85.0)	0.709	0.400
4. Do you think it of getting HIV/AIDS from daily living and study contact (no)	1351 (84.3)	1159 (84.7) 192 (82.1)		1.079	0.299
5. Do you agree that the risk of contracting and transmitting HIV/AIDS can be reduced by consistent and correct condom use (yes)	1482 (92.5)	1284 (93.9)	198 (84.6)	24.642	< 0.001
6. Does the use of new drugs (e.g., methamphetamine, ecstasy, ketamine, etc.) increase the risk of HIV infection (yes)	1381 (86.2)	1197 (87.5)	184 (78.6)	13.212	< 0.001
7. Should you seek HIV testing and counselling after high risk behaviours (needle sharing/unsafe sex, etc.) (yes)	1497 (93.4)	1291 (94.4)	206 (88.0)	13.102	< 0.001
8. Are the rights of people living with HIV to marry/employ/enroll in school protected by our laws (yes)	1296 (80.9)	1129 (82.5)	167 (71.4)	16.110	< 0.001
9. Do you agree that you need to use condoms even if you have sex with someone you know (yes)	1381 (86.2)	1178 (86.1)	203 (86.8)	0.069	0.793

The average score of knowledge about HIV/AIDS out of 9 was 7.43 ± 1.63. 527 (32.9%) participants answered all 9 items correctly. Compared to other groups, the lower HIV/AIDS-related knowledge score was significantly performed in female students (7.14 ± 2.26), ethnic minority (6.96 ± 2.30), married (4.89 ± 3.04), those who lived on over 3000 Yuan per month (6.23 ± 2.81), those who reported bisexual (6.28 ± 2.69), respectively (Table 3).

**Table 3:** Comparisons of demographics variables associated with level of HIV-related knowledge among the study participants (n = 1602)

Variables	Category	Score (M ± SD)	*T* or *F*	*P*
Gender	Male	7.63 ± 1.60	4.044	< 0.001
	Female	7.14 ± 2.26		
Grade	Freshmen	7.45 ± 1.75	3.969	0.008
	Sophomore	7.51 ± 1.75		
	Junior	7.81 ± 1.39		
	Senior	7.45 ± 2.01		
Ethnicity	Han	7.62 ± 1.64	4.550	< 0.001
	Minority	6.96 ± 2.30		
Marriage	Unmarried	7.67 ± 1.55	13.218	< 0.001
	married	4.89 ± 3.04		
Monthly income	Under 1000	7.47 ± 1.72	24.757	< 0.001
	1000 ~	7.72 ± 1.49		
	2000 ~	7.67 ± 1.78		
	Above 3000	6.23 ± 2.81		
Sexual orientation	Heterosexual	7.72 ± 1.50	51.513	< 0.001
	Homosexual	6.81 ± 2.33		
	Bisexual	6.28 ± 2.69		

### Source of HIV prevention services

The top three ways of acquiring HIV/AIDS-related Knowledge for the participants were rated as broadcasting, the internet and books and newspapers (77.5, 76.7, and 70.2%, respectively); about 43% of female participants considered health education sessions as an additional way to access HIV/AIDS-related knowledge. Less than 60% of participants reported that they had received HIV prevention services; even more, only 15% had tested for HIV (Table 4).

**Table 4:** Utilization of HIV prevention services among the study participants (n = 1602)

Services	Total N = 1602	Male (%) 1368	Female (%) 234
Ways to acquire HIV/AIDS-related knowledge			
Broadcasting	1241 (77.5)	1086 (79.4)	155 (66.2)
Books and newspapers	1124 (70.2)	986 (72.1)	138 (59.0)
Lectures	903 (56.4)	790 (57.7)	113 (48.3)
Medical personnel	895 (55.9)	774 (56.6)	121 (51.7)
Family and friends	781 (48.8)	673 (49.2)	108 (46.2)
Internet	1228 (76.7)	1032 (75.4)	196 (83.8)
Health education courses	961 (60.0)	807 (59.0)	154 (65.8)
Others	77 (4.8)	63 (4.6)	14 (6.0)
Willingness to acquire HIV/AIDS-related knowledge throughout various ways			
Consultation	672 (41.9)	611 (44.7)	61 (26.1)
Playing HIV-related video	904 (56.4)	791 (57.8)	113 (48.3)
Posters propaganda	613 (38.3)	541 (39.5)	72 (30.8)
Brochure	562 (35.1)	483 (35.3)	79 (33.8)
Broadcasting	389 (24.3)	321 (23.5)	68 (29.1)
Books and newspapers	334 (20.8)	275 (20.1)	59 (25.2)
Internet	801(50.0)	658 (48.1)	143 (61.1)
Health education courses	502 (31.3)	402 (29.4)	100 (42.7)
Others	29 (1.8)	22 (1.6)	7 (3.0)
Willingness to participant in publicity HIV/STDs prevention service			
Yes	1289 (80.5)	1103 (80.6)	186 (79.5)
No	188 (11.7)	165 (12.1)	23 (9.8)
Unclear	125 (7.8)	100 (7.3)	25 (10.7)
Ever had received HIV prevention service			
Yes	900 (56.2)	786 (57.5)	114 (48.7)
No	702 (43.8)	582 (42.5)	120 (51.3)
Had HIV testing before			
Yes	241 (15.0)	208 (15.2)	33 (14.1)
No	1361 (85.0)	1160 (84.8)	201 (85.9)

### Risk sexual behaviours and factors correlated

Of 1602 university students who reported ever having had sexual intercourse, having multiple sexual partners, inconsistent condom use and both in the past 6 months were 37.3, 35, 20%, respectively. More than half of participants had their sex before the age of 18; 48% of them reported no use of condom during their sexuality; 52.5% of them reported having multiple sexual partners. Of the 47 male students who reported being homosexual, 66% had sex with two or more sexual partners. Of the 106 male students who reported being bisexual, 95% had multiple sexual partners with the same sex or opposite sex and 75.7% reported no condom use in the past 6 months. Table 5 shows the link between demographics and risk sexual behaviours among the respondents. Consistent condom use over the last 6 months was higher in the superior grades (junior and senior) compared to the lower grades (freshmen and sophomore). Over 80% of students who lived on over 3000 Yuan per month had multiple sexual partners and about 70% inconsistent condom use. About 65% of students who reported being homosexual had multiple sexual relationship and approximately 77% of students who reported being bisexual did not consistently use condoms. There was a statistically significant difference (*P* < 0.05).

**Table 5:** Comparisons of demographics variables associated with risk sexual behaviours among the study participants

Characteristic	Number of sexual partner			Condom use during the last six months		
	Single	Multiple	*P*	Consistent	Inconsistent	*P*
Gender						
Male	859 (62.8)	509 (37.2)	0.809	891 (65.1)	477 (34.9)	0.560
Female	145 (62.0)	89 (38.0)		157 (67.1)	77 (32.9)	
Grade						
Freshmen	237 (61.9)	146 (38.1)	0.855	234 (64.5)	149 (38.9)	0.001
Sophomore	332 (62.1)	203 (37.9)		329 (61.5)	206 (38.5)	
Junior	258 (64.5)	142 (35.5)		284 (71.0)	116 (29.0)	
Senior	177 (62.3)	107 (37.7)		201 (70.8)	83 (29.2)	
Ethnicity						
Han	923 (63.7)	526(36.3)	0.009	949 (65.5)	500 (34.5)	0.846
Minority	81 (52.9)	72 (47.1)		99 (64.7)	54 (35.3)	
Marriage						
Unmarried	991 (64.4)	548 (35.6)	< 0.001	1036 (67.3)	503 (32.7)	< 0.001
Married	13 (20.6)	50 (79.4)		12 (19.0)	51 (81.0)	
Monthly income						
Under 1000	226 (59.0)	157 (41.0)	< 0.001	239 (62.4)	144 (37.6)	< 0.001
1000 ~ 2000	669 (69.5)	293 (30.5)		673 (70.0)	289 (30.0)	
2001 ~ 3000	91 (58.7)	64 (41.3)		106 (68.4)	49 (31.6)	
Above 3000	18 (17.6)	84 (82.4)		30 (29.4)	72 (70.6)	
Sexual orientation						
Heterosexual	976 (69.5)	429 (30.5)	< 0.001	985 (70.1)	420 (29.9)	< 0.001
Homosexual	22 (34.9)	41 (65.1)		33 (52.4)	30 (47.6)	
Bisexual	6 (4.5)	128 (95.5)		30 (22.4)	104 (77.6)	

The model I ‘the number of sexual partner (single = 0; multiple = 1)’, the model II ‘whether or not consistent condom use during the past six months (No = 0; Yes = 1)’, independent variable (including demographic characteristics, level of HIV/AIDS-related knowledge, self-perceived risk of HIV infection and age of first sexual intercourse) were performed for multivariable logistic regression analysis. The results of the analysis were presented in Table 6. Participants who had two or more sexual partners were high-grade students (AOR = 1.56; 95% CI 1.12, 2.18 for sophomore; AOR = 1.84; 95% CI 1.28, 2.65 for junior; AOR = 2.07; 95% CI 1.38, 3.11 for senior), those who lived on over 3000 Yuan per month (AOR = 4.19; 95% CI 2.17, 8.11), reported being homosexual (AOR = 3.92; 95% CI 2.17, 7.06) and bisexual (AOR = 33.22; 95% CI 13.11, 84.15). Sufficient HIV/AIDS-related knowledge (AOR = 1.93; 95% CI 1.16, 3.19), Self-perceived risk of HIV infection (AOR = 2.19, 95% CI 1.47, 3.27), age at first sexual intercourse (AFSI) over 18-year-old (AOR = 2.47; 95% CI 1.88, 3.24) were positively associated with consistent condom use during the last 6 months. Participants were likely not to use condoms consistently if they reported being bisexual (AOR = 0.27; 95% CI 0.16, 0.44), with a monthly income of over 3000 Yuan (AOR = 0.50; 95% CI 0.28, 0.91). Statistical significance was set at *P* = 0.05.

**Table 6:** Multiple logistic regression analysis predicting factors associated with risk sexual behaviours among the study participants

Independent variable	Model I		Model II	
	AOR (95% CI)	*P*	AOR (95% CI)	*P*
Demographic characteristics				
Gender				
Female	1.00 (ref)	0.674	1.00 (ref)	0.132
Male	1.08 (0.75, 1.55)		0.74 (0.50, 1.10)	
Grade				
Freshmen	1.00 (ref)		1.00 (ref)	
Sophomore	1.56 (1.12, 2.18)	0.008	0.86 (0.59, 1.12)	0.207
Junior	1.84 (1.28, 2.65)	0.001	1.02 (0.71, 1.48)	0.912
Senior	2.07 (1.38, 3.11)	< 0.001	1.16 (0.76, 1.77)	0.488
Ethnicity				
Han	1.00 (ref)	0.170	1.00 (ref)	0.147
Minority	1.34 (0.88, 2.03)		1.41 (0.89, 2.24)	
Marriage				
Unmarried	1.00 (ref)	0.171	1.00 (ref)	0.134
Married	0.52 (0.20, 1.33)		0.51 (0.21, 1.23)	
Monthly income				
Under 1000	1.00 (ref)		1.00 (ref)	
1001 ~ 2000	0.79 (0.59, 1.06)	0.110	1.16 (0.86, 1.57)	0.329
2001 ~ 3000	1.10 (0.71, 1.70)	0.677	1.35 (0.83, 2.18)	0.229
Above 3000	4.19 (2.17, 8.11)	< 0.001	0.50 (0.28, 0.91)	0.022
Sexual orientation				
Heterosexual	1.00 (ref)		1.00 (ref)	
Homosexual	3.92 (2.17, 7.06)	< 0.001	0.60 (0.32, 1.13)	0.114
Bisexual	33.22 (13.11, 84.15)	< 0.001	0.27 (0.16, 0.44)	< 0.001
Level of HIV-related Knowledge				
Insufficient (< 6 scores)	1.00 (ref)	0.210	1.00 (ref)	0.011
Sufficient (≥ 6 scores)	0.73 (0.44, 1.20)		1.93 (1.16, 3.19)	
Risk perception of HIV/STDs				
No	1.00 (ref)	< 0.001	1.00 (ref)	< 0.001
Yes	0.37 (0.24, 0.56)		2.19 (1.47, 3.27)	
Age of first sexual activity				
≤ 18	1.00 (ref)	0.001	1.00 (ref)	< 0.001
> 18	0.32 (0.25, 0.42)		2.47 (1.88, 3.24)	

Model I: number of sexual partner; single = 0, multiple = 1;

Model II: whether or not condom use during the last six months; No = 0, Yes = 1

*CI* confidence interval

## Discussion

AIDS is now the second leading cause of death among adolescents globally [32]. The trend of the HIV epidemic among young students is growing significantly and university students, as pillars of our nation, are the heart of HIV prevention and care. We found that of the participants surveyed, around 9% had ever had sex (11% for male students and 4% for female students, respectively). This was in line with another study conducted in 2017, Henan Province [33], but much lower than a study indicating that 18.7% reported having ever had sexual intercourse [8]. Due to privacy or sensitive issues related to this survey, the outcome of the study is slightly less than the actual outcome. Participants who reported having ever had sexual intercourse, risk sexual behaviours, are more vulnerable to HIV infection and are likely to come into contact with the general population, such as heterosexual female students. Inconsistent condom use and multiple sexual partners are recognized common types of risk sexual behaviours. Early AFSI, sufficient HIV/STDs-related knowledge, sexual orientation reported non-heterosexual, students with high standard living, high self-perceived risk of HIV infection were identified by associated factors, affecting condom use and sexual partners among university students.

Xu’s study indicated that knowledge of HIV/AIDS was not effective in changing people's behaviour, with no significant positive correlation between adequate HIV/AIDS-related knowledge and safe sex [22]. In our study, however, students with sufficient HIV/AIDS-related knowledge were more likely to adhere to condom use during the last 6 months. Although most students knew that condom use could reduce the risk of HIV/STDs infection, having more easily access to sexual contents online tend to engage in risk sexual behaviours [34]. A significant correlation between awareness of HIV/AIDS-related knowledge and sexual partner was not found. But the risk of HIV infection should be addressed by those with a low level of knowledge. We also found that 45% of participants thought HIV is far from their lives (21% of 47 gay university students) and nearly half of 47 MSM students did not know the male-to-male transmission is the main mode of transmission of HIV infection among adolescents. These indicated that education on HIV prevention and control, involving sex and reproductive health (SRH), is urgent. Sex education is an important entry for HIV prevention and control [35] and strengthens the breadth of knowledge, especially in precautions on HIV/STDs and attitudes about homosexuality and HIV infection [30], to reduce the risk of sexual behaviour and other sexually transmitted infections. In addition, we should develop new media platforms such as websites and apps that can attract the attention of young students and use these resources to guide them well.

Sexual orientation, an important factor, has a significant impact on sexual behaviours among young students [36]. Non-heterosexual university students preferred to engage in risk sexual behaviours. Compared to homosexuals, bisexuals were less likely to use condoms and have multiple sexual partners, a finding identified in another study [37]. Meanwhile, non-heterosexuals still suffer from strong social pressures such as discrimination, even though social acceptance of the lesbian, gay, bisexual and transgender (LGBT) community is slowly increasing in China [38]. This may result in the expansion of the HIV epidemic among women as a whole. On one hand, some male students reported being bisexual concealed their sexual identity, even dating female students and having sex with them. Furthermore, once graduated from university and moved into society, they may be compelled to marry straight women under the pressure of family and parents, social environment [39], which expanded a more HIV epidemic. On the other hand, discrimination and stigma from peer and society make HIV testing challenging. Only 15% had been tested for HIV in our study, which was identified with the result reported by another study [40]. As of 2018, about 30% of PLWH in China were not detected [41]. Many MSM are not aware of their ability to contract with their sexual partners, which increases the risk of HIV transmission. These challenges must be responded and addressed through aggressive actions.

We also found that over half of students ever having sex reported having had sexual contact before the age of 18, which much higher than that of other results [8, 17]. This indicates an increasing trend for young students to have their sexual debut at an earlier age, which is in line with Zou’s study [42]. Moreover, they are more likely to engage in risk sexual behaviours. This may be due to the lack of sexual knowledge on sex and reproductive health (including HIV/AIDS-related knowledge) they have prior to their sexual debut. Although sex education has been introduced into the secondary school curriculum since 1988, only a small number of schools (nearly half of the universities) offered it to students [30]. In the current crisis where the HIV epidemic among young students continues to grow, scaling up sex education is an important intervention [43]. Only the top third of students in China are eligible to attend university [30], and for the remaining young people who do not attend university, there are real challenges in targeting this group with sex education.

In line with another study [44], university students with high living costs, especially over 3000 Yuan per month, prefer to less condom use and engage in having sex with multiple sexual partners. This may be related to the family environment in which they grow up. In China, students living on more than 3000 Yuan tend to come from relatively wealthy families. Yan’s study has yielded the result that female students who reported having had sex were more likely to be from richer families [45]. However, there is limited literature on the mechanisms underlying the relationship between family socioeconomic status and sexual behaviour of children. It is noteworthy that family education and the role of parents are as important as school education in the development of adolescents, guiding and helping in a correct view of sexuality and marriage. In an era of rapid economic development in contemporary China, parents of high-income families may spend most of their energy on work, with little regard for the education and supervision of their children, especially when children are in their rebellious teenage years. An early study has documented that adolescents lacking parental monitoring were more likely to engage in risk sexual behaviours [46]. Briefly, communication between mothers and daughters help in safe sex among female students with low levels of HIV/STDs-related knowledge compared to male students [47]. Thus, initiation efforts to sex education should focus on students with a high standard of living and consumption, including their parents.

Various limitations in this study must be highlighted. Firstly, uneven distribution of the study participants by gender and grade may affect the reliability of the comparison results. Secondly, as the nature of the cross-sectional survey and the privacy and sensitivity of the questionnaire, information gathered from the questionnaires answered by the study participants may be subject to information bias. Besides, students from one university in Henan Province was selected to participate in the study. In the future study, a cross-sectional survey consisting of several universities and schools in Henan should be conducted to further identify factors associated with risk sexual behaviours among sexually active young students.

## Conclusions

Our study described the demographic characteristics of university students in Henan who reported having ever had sex, including their level of knowledge about HIV/AIDS and utilization of HIV prevention services, identified factors associated with their risk sexual behaviours, and specified high-risk groups at risk of HIV/STDs infection. Inconsistent condom use during the last 6 months as well as having multiple sexual partners as commonly risk sexual behaviours are frequent among university students, especially gay male students, bisexual male students. Non-heterosexual university students become key to the HIV epidemic among the university population. There are currently many shortcomings in HIV prevention work in schools that need to be reinforced. Targeted services for key populations among university students should be widely implemented in universities and other technical schools, secondary schools, including educating parents about the sexual behaviour of their children.

## Data Availability

The dataset analyzed during the current study is available from the corresponding author on reasonable request.

## Abbreviations

HIV/AIDS: Human immunodeficiency virus/acquired immunodeficiency syndrome
PLWH: People living with HIV
MSM: Men who have sex with men
MSWM: Men who have sex with women and men
STDs: Sexually transmitted diseases
UAI: Unprotected anal intercourse
UVI: Unprotected vaginal sex
AFSI: Age at first sexual intercourse
LGBT: Lesbian, gay, bisexual and transgender
CI: Confidence interval
AOR: Adjusted odds ratio
SD: Standard deviation
ANOVA: Analysis of variance
